# Strategies for optimizing maternal nutrition to promote infant development

**DOI:** 10.1186/s12978-018-0534-3

**Published:** 2018-06-22

**Authors:** K. Michael Hambidge, Nancy F. Krebs

**Affiliations:** Section of Nutrition, Department of Pediatrics, School of Medicine, University of Colorado Anschutz Medical Campus, 12700 E 19th Ave, MS C-225, Aurora, CO 80045 USA

**Keywords:** Maternal, Nutrition, Multiple micronutrients, Small-quantity lipid-based nutrition supplements, Pregnancy outcomes

## Abstract

**Background:**

The growing appreciation of the multi-faceted importance of optimal maternal nutrition to the health and development of the infant and young child is tempered by incompletely resolved strategies for combatting challenges.

**Objective:**

To review the importance of maternal nutrition and strategies being employed to optimize outcomes.

**Methods:**

Selected data from recent literature with special focus on rationale for and currently published results of maternal nutrition supplements, including lipid based nutrition supplements.

**Results:**

1) An impelling rationale for improving the maternal and in utero environment of low resource populations has emerged to achieve improved fetal and post-natal growth and development. 2) Based partly on population increases in adult height over one-two generations, much can be achieved by reducing poverty. 3) Maternal, newborn and infant characteristics associated with low resource environments include evidence of undernutrition, manifested by underweight and impaired linear growth. 4) Apart from broad public health and educational initiatives, to date, most specific efforts to improve fetal growth and development have included maternal nutrition interventions during gestation. 5) The relatively limited but real benefits of both iron/folic acid (IFA) and multiple micronutrient (MMN) maternal supplements during gestation have now been reasonably defined. 6) Recent investigations of a maternal lipid-based primarily micronutrient supplement (LNS) have not demonstrated a consistent benefit beyond MMN alone. 7) However, effects of both MMN and LNS appear to be enhanced by commencing early in gestation.

**Conclusions:**

Poor maternal nutritional status is one of a very few specific factors in the human that not only contributes to impaired fetal and early post-natal growth but for which maternal interventions have demonstrated improved in utero development, documented primarily by both improvements in low birth weights and by partial corrections of impaired birth length. A clearer definition of the benefits achievable by interventions specifically focused on correcting maternal nutrition deficits should not be limited to improvements in the quality of maternal nutrition supplements, but on the cumulative quantity and timing of interventions (also recognizing the heterogeneity between populations). Finally, in an ideal world these steps are only a prelude to improvements in the total environment in which optimal nutrition and other health determinants can be achieved.

## Background

With the support of the Bill and Melinda Gates Foundation and within the infrastructure provided by the NICHD Global Network for Women’s and Children’s Health Research, the University of Colorado School of Medicine is currently collaborating with four Global Network sites including JN Medical Center in KLE University, Belagavi to evaluate the effects of maternal nutrition supplements commenced prior to conception on fetal and post-natal growth and development [1]. The principal purpose of this paper is to provide relevant background to this trial by reviewing the results of recent randomized controlled studies of maternal nutrition supplements on newborn anthropometry as a measure of fetal growth.

Increasingly, the rapidly advancing epidemic of overweight / obesity in the developing world, together with its attendant complications of diabetes, hypertension, and other metabolic manifestations of non-communicable disease (NCD), are attracting attention and are of great public health concern. Moreover, ‘thrifty gene’ and ‘developmental origins of health and disease’ (DoHaD) hypotheses have firmly established that fetal growth impairment and associated metabolic adaptations are a common prelude to later overweight / obesity and NCD [2, 3]. Hence, the outstanding role of fetal development to successful short and long-term successful pre- and post-natal outcomes continues to gain recognition. Epidemiologically, impaired fetal growth is manifest by low birth weight (LBW) attributable to both preterm birth and small-for-gestational-age (SGA), which is the leading cause of neonatal and early infant mortality in low resource populations [4]. More than 20 million infants in low-middle income countries are born SGA each year with the highest incidence in southwestern India [5]. Impaired linear growth as well as SGA predict suboptimal post-natal growth including stunting at 2 years of age [6, 7] and will be a focus of this review [8]. Maternal under-nutrition is associated with LBW especially that attributable to SGA [9] and with impaired fetal linear growth and is high on the global health agenda [1].

As with post-natal growth and development, long-term population-wide solutions to improve fetal growth and development depend on population-wide economic and social improvements. Meanwhile and, indeed, for the foreseeable future, components of the adverse environment are likely to require improvements on an individual basis. Maternal nutrition is an outstanding example. The phenotypic variations, both short and long-term, related to diet and to nutritional status, and the changes associated with nutrition interventions are legion, and our understanding of these continues to expand exponentially. However, newborn anthropometric data have durable associations with physiological and pathophysiological outcomes in post-natal life. These provide the mainstay of this paper that reviews strategies to improve maternal nutrition with the primary goal of promoting fetal and infant growth and development.

## Strategies for improving maternal nutrition

### Public Health Strategies

The most effective strategies, though perhaps most challenging, are those that address food deficits of the entire community / population, or at least those of females at all ages. Recent experience in Brazil, for example, has demonstrated how economic improvements for the population are followed quickly by rapid closure of the gap between low and high socio-economic groups in the incidence of stunting [10]. Cross-population differences in height are related at least primarily to environmental factors and changes in population heights for adult men and women provide invaluable insights into changes in the environment, including nutrition, over successive generations [11]. In contrast to major increases in height in some previously resource poor settings, there has been little change in the relatively low adult heights in South Asia over the past century. However, there is recent improvement in heights of young children with absolute gains in height-for-age (HAZ) Z-scores for 141 developing countries being greatest in South Asia, with mean HAZ increasing by 0.4 cm per decade between 1985 and 2011 [12]. The relevance of these findings to this paper is that these changes must be attributable to changes in the environment, including, but not limited to diets, and that these changes must have impacted linear growth of the fetus and the infant and toddler. Advantages associated with increases in height include fewer pregnancy complications, expected higher education, earnings, and greater longevity with lower risk of cardiovascular and respiratory disease. Population-wide improvement in short stature requires improved environment including nutrition during development especially in utero and during the first 2–3 years of post-natal life and subsequently during adolescence. If nutrition resources are limited, priority should be given to the nutrition of females from birth through the reproductive years, which may entail cultural changes and empowerment of women [13–16].

At a more pragmatic but still ambitious level, bio-fortification of staple crops and fortification of food staples during food processing offer potential “passive” strategies to improve micronutrient status. For example, recent research with pearl millet, biofortified with zinc and iron, demonstrated good absorption and substantial contributions to meet the physiologic requirements for young children [17]. Experience with iron biofortified crops, including pearl millet, rice, and beans, has been encouraging for improvements in iron status in women of reproductive age, with the greatest efficacy in those who were iron deficient at baseline [18]. Trials to examine functional outcomes are now warranted to assess feasibility for scaling up bio-fortification as a realistic preventive strategy. A potentially very important single micronutrient fortification is folate fortification of a food staple, most conveniently flour for populations that utilize a central communal mill [19]. Currently, there is a paucity of data on the prevalence of NTD in many low-middle income countries [20] and lack of adequate assessment of the effectiveness of national folate fortification programs.

### Diet

Dietary improvements are theoretically the most appealing and potentially sustainable of strategies to improve and maintain maternal nutrition. They are also the most challenging as they depend on economic factors, cultural influences, education, and broader ecological factors including agriculture. Additional challenges may surface during gestation ranging from changes in appetite, nausea, and vomiting in early gestation to fear of obstructed labor due to cephalo-pelvic disproportion.

A recent overview of systematic reviews focused specifically on nutrition-specific interventions targeting LBW as the outcome. This review included results of multiple randomized controlled trials (RCTs), with 16 reviews of high quality [21]. Overall, improvements in women’s nutritional status were associated with positive effects on LBW, SGA, and PTB. Specifically, nutrition education plus multi-micronutrient (MMN) supplements, oral supplements of Vitamin A, low dose calcium, and zinc during pregnancy decreased the risk of LBW.

Publications in this supplement include one by Drs S. Mastiholi et al., reporting their own recent experience with identifying risks of nutritional deficits specifically in N. Karnataka [22]. Their locally developed food composition data base, including a wealth of regional recipes, has been facilitated by the Women First preconception trial which supported the acquisition of dietary data for women during the first trimester of pregnancy [23].

### Maternal nutrition supplements

Supplements during pregnancy, typically starting in the second trimester are popular globally, being easy to take and, except for more comprehensive supplements, relatively affordable. The supplements to be considered here are iron/folic acid (IFA)**,** MMN, and more comprehensive nutrition supplements, the most widely used of which are small quantity lipid-based nutrition supplements especially designed for pregnancy/lactation.

### Iron folate supplements (IFA)

are recommended globally by WHO and are now quite typically included in national guidelines for low middle-income countries. Limitations to their use include insufficient or absent supplies in local health centers and poor compliance in their use. However, this concern may not apply to the catchment area for KLE’s Jawaharlal Nehru Medical College (KLE-JNMC) where local health services appear effective in distributing and achieving compliance with utilizing ante-natal IFA. Despite this, anemia remains a major public health challenge in India [24]. Incompletely answered questions include the extent to which the anemia is attributable to iron deficiency and whether a modest reduction in the current standard of care for pregnancy of 60 mg iron/day would be better accepted and, therefore, more efficacious. With respect to the goals of this paper, there have been individual reports of improved birth size attributable to IFA but these have not been confirmed by meta-analysis [25].

### Multiple micronutrients (MMN)

The 2016 WHO recommendations did not discourage but did not make a recommendation for the use of MMN as a routine part of antenatal care [26]. Subsequently, there have been comprehensive reviews and meta-analyses [21, 27, 28] which confirmed that daily MMN reduced the risk of LBW and SGA in comparison with IFA alone. Additional findings included a reduction in mortality for female neonates and greater reductions in LBW, preterm birth and SGA for women with indicators of malnutrition during pregnancy. No harmful effects of MMN were revealed.

### Lipid-based nutrient supplement (LNS)

(Nutriset, Malauney, France**):** LNS refers to a 20 g composite of MMN with polyunsaturated lipids (4.9 g linoleic and 0.5 g alpha-linolenic) providing a lipid-based supplement. Composition typically includes dried skimmed milk, soybean and peanut extract, sugar, maltodextrin, stabilizers and emulsifiers. For pregnant and lactating women, various minor modifications of the micronutrient composition have been made. Each sachet provides 118 kcal and 2.6 g protein. LNS is a modification of Nutributter. With newborn length as one outcome, LNS or a similar preparation has been compared with MMN in four RCT [29–32]. The increase in effect size for length-for-age Z-score (LAZ) was moderate and statistically significant for the study in Burkina Faso that used what may be considered a forerunner of LNS [29]. For the other trials, effect sizes on LAZ were low and not statistically significant. Subgroup analyses revealed additional insights. In Ghana, parity was an important modifier, with a relatively high increase in LAZ of 9 mm for primiparous women [31]. In contrast, in Burkina Faso the LNS equivalent only had a significant effect on birth length in multigravida women. Additionally, the intervention was more efficacious in women with low baseline BMIs including an 11–12 mm increase in birth length, 111 g increase in weight, and ~ 50 g increase in placental weight [29]. Seasonality at this site has been reported to be a major modifier of the effect of LNS on birth length [33]. A retrospective study in Gambia did not reveal any increase in outcome measures of birth size for LNS vs MMN [34].

Comparison of LNS with IFA supplements included modest but statistically significant higher birth lengths (2 mm) and LAZ (0.10) for the LNS group in Bangladesh [32], whereas no impact of LNS, IFA or MMN was observed in Malawi for any birth outcomes [30]. In Ghana, the offspring of the mothers who continued to receive the LNS through 6 months post-partum and whose offspring received the corresponding supplement from 6 months of age had significantly higher lengths, LAZ, and weight-related measures than either the MMN or IFA groups at 18 months of age [35]. Similar results were observed in Bangladesh [36].

Potentially pertinent to both the use and future research on maternal nutrition supplements with both LNS and MMN, has been the correlation of birth length related outcomes with cumulative dose of LNS in pregnancy; this is largely related to gestational age at commencement of the supplement [37]. More specifically, the data indicated an advantage in commencing the supplement in the 1st trimester. Other studies have also indicated advantages of starting supplements in the 1st trimester including a trial of prenatal micronutrient and food supplementation in Bangladesh [38].

### Nutrition supplements commencing prior to conception

The formidable challenge of early identification of conception and, therefore, of commencing nutrition interventions early in gestation are but one of the attractions of starting supplements prior to conception. This, in turn, implies measures on a broad public health scale to ensure adequate nutrition of all females of child-bearing age. Additional incentives for preconception nutrition supplements include the potential value to the fetus of correcting maternal weight deficits prior to conception. Additionally, emerging evidence supports the occurrence of adverse epigenetic changes in the embryo that may be correctable with improved nutrition [39, 40]. For single nutrients, preconception folate supplements for prevention of neural tube defects are of outstanding importance. Completed trials of more comprehensive nutrition supplements commencing prior to conception are very limited. One included an ambitious intervention that provided food snacks, including leafy vegetables, fruit and milk. Results were consistent with a positive effect on birth weight in the sub-group who commenced the intervention at least 3 months prior to conception [41]. For the past 5 yrs. the National Institute of Child Health and Human Development (NICHD) Global Network for Women’s and Children’s Health Research has participated in a trial to test the benefit of a comprehensive nutrition supplement commencing ≥3 months prior to conception vs commencing at ~ 12 wks gestation vs not at all. As noted above, KLE-JNMC is one of four network sites participating in this trial. Results of newborn anthropometry will be available later in 2018. It is anticipated that the results of this complex study will provide new insights into the value of maternal nutrition supplements commencing either early in gestation or prior to conception in multiple sites with heterogeneous cultures and diets across three continents [1].

Parental, especially maternal height, and body mass index (BMI) have strong associations with fetal and infant growth and subsequently with stature later in childhood [42]. While the effects of low maternal BMI can reasonably be expected to be minimized with relatively short term improved maternal nutrition prior to conception [43], maternal height cannot be changed with maternal nutrition improvements in her adult life. As a minimum, correction of the deficits in linear growth would have required improvements in maternal environment including nutrition environment in utero and, to some extent, during her first 2 years of post-natal life. Moreover, there is convincing evidence for the relevance of intergenerational effects at least in some populations which cannot always be attributed directly to persistence of the same poor environment across generations [44]. As yet there are more questions than answers on the extent of the role of potentially modifiable epigenetic changes in determining fetal and infant growth; however, there is now encouraging evidence that beneficial changes may occur with improvement in maternal nutrition [40]. This is a priority area for more extensive research. Recognizing that reduction in stunting is likely to be achieved by increments and by more than one pathway gives more meaning to the modest gains that are being achieved with just one intervention over relatively brief time intervals.

The greatest challenge for fetal and post-natal development in India currently is the very high incidence of SGA [5]. Frequently, this has an unusual presentation, termed the ‘thin-fat baby,’ in which infants are born with diminished abdominal viscera and muscle mass but preserved body fat. Manifestations of the syndrome persist into adulthood especially with a high incidence of insulin resistance and obesity. An epigenetic, multigenerational cause has been proposed for this syndrome which is not amenable to short term maternal nutrition interventions [45, 46].

## Conclusions

- Inadequate maternal nutrition has been identified convincingly as a specific adverse environmental factor that contributes to impaired fetal growth in low resource populations.
- Ideal strategies for prevention / management require adequate quality food and education but in practice nutrition supplements are necessary.
- Iron-folate (IFA) is a cornerstone of maternal nutrition management but does not impact fetal growth. Apart from exceptional circumstances, other single micronutrient deficiencies are covered with MMN.
- Multi-micronutrient supplements (MMN) are of demonstrated but limited value for fetal growth.
- Effects of the current generation of lipid nutrition supplements (LNS) have not exceeded those of MMN.
- Large effects of both MMN and LNS have been documented in certain sub-group analyses, but findings are not consistent.
- Effects may also be greater when supplements are commenced early in gestation.
- In practice, optimal maternal nutrition will be achieved with a combination of educated use of the local foods available supplemented as necessary with MMN or more complete nutrient supplements, the latter being advantageous if the macronutrient supply is deficient in quantity or quality.

Research Agenda:

Important directions for research include:

- Development and testing of new generations of nutrition supplements that are intended to provide adequate quantities of nutrients.
- Testing longer-term nutrition supplements for women. Unfortunately, it is impractical to test nutrition interventions over an entire generation. However, it is reasonable, if challenging, to commence interventions prior to conception. Theoretical advantages of this ‘early start’ include increasing the duration over which maternal nutrition deficiencies can be corrected and ensuring optimal maternal nutrition by the time of conception, achieving coverage with improved maternal nutrition during the peri-conception period and first trimester.
- Testing the impact of improvements in girls’ nutritional status during adolescence on later reproductive outcomes.
- Examining potential for improved maternal nutrition during a reproductive cycle to have other health benefits, e.g. metabolic health, cognitive function.

## Abbreviations

BMI: Body mass index
DOHaD: Developmental origins of health and disease
IFA: Iron/Folic Acid
KLE-JNMC: KLE Jawaharlal Nehru Medical College
LAZ: Length-for-age Z score
LBW: Low birth weight
LNS: Lipid-based nutrition supplement
MMN: Multiple micronutrient
NICHD: National Institute of Child Health and Human Development
PTB: Preterm birth
SGA: Small-for-gestational-age
